# Pilot clinical study of ascorbic acid treatment in cardiac catheterization

**DOI:** 10.1093/jrr/rrz038

**Published:** 2019-06-28

**Authors:** Lue Sun, Tomonori Igarashi, Ryoya Tetsuka, Yun-Shan Li, Yuya Kawasaki, Kazuaki Kawai, Haruhisa Hirakawa, Koji Tsuboi, Asako J Nakamura, Takashi Moritake

**Affiliations:** 1 Health Research Institute, Department of Life Science and Biotechnology, National Institute of Advanced Industrial Science and Technology (AIST), Central 6, 1-1-1 Higashi, Tsukuba, Ibaraki, Japan; 2 Department of Radiation Biology, Faculty of Medicine, University of Tsukuba, 1-1-1 Tennodai, Tsukuba, Ibaraki, Japan; 3 Department of Radiological Health Science, Institute of Industrial Ecological Sciences, University of Occupational and Environmental Health, Japan, 1-1 Iseigaoka, Yahatanishi-ku, Kitakyushu, Fukuoka, Japan; 4 Iwamoto Hospital, 1-2-8 Shimoishida, Kokuraminami-ku Kitakyushu, Fukuoka, Japan; 5 Department of Occupational Toxicology, Institute of Industrial Ecological Sciences, University of Occupational and Environmental Health, Japan, 1-1 Iseigaoka, Yahatanishi-ku, Kitakyushu, Fukuoka, Japan; 6 Department of Biological Sciences, College of Science, Ibaraki University, 2-1-1 Bunkyo, Mito, Ibaraki, Japan; 7 Department of Environmental Oncology, Institute of Industrial Ecological Sciences, University of Occupational and Environmental Health, Japan, 1-1 Iseigaoka, Yahatanishi-ku, Kitakyushu, Fukuoka, Japan; 8 Department of Cardiology, Social Insurance Nogata Hospital, 1-1 Susakimachi, Nogata, Fukuoka, Japan

**Keywords:** interventional radiology, medical exposure, γH2AX, ascorbic acid, radiation protection, glutathione

## Abstract

Clinical radiodiagnosis and radiotherapy sometimes induce tissue damage and/or increase the risk of cancer in patients. However, in radiodiagnosis, a reduction in the exposure dose causes a blockier image that is not acceptable for diagnosis. Approximately 70% of DNA damage is induced via reactive oxygen species and/or radicals created during X-ray irradiation. Therefore, treatment with anti-oxidants and/or radical scavengers is considered to be effective in achieving a good balance between image quality and damage. However, few studies have examined the effect of using radical scavengers to reduce radiation damage in the clinical setting. In this study, we administrated 20 mg/kg ascorbic acid (AA) to patients before cardiac catheterization (CC) for diagnostic purposes. We analyzed changes in the number of phosphorylated H2AX (γH2AX) foci (a marker of DNA double-strand breaks) in lymphocytes, red blood cell glutathione levels, blood cell counts, and biochemical parameters. Unfortunately, we did not find satisfactory evidence to show that AA treatment reduces γH2AX foci formation immediately after CC. AA treatment did, however, cause a higher reduced/oxidized glutathione ratio than in the control arm immediately after CC. This is a preliminary study, but this result suggests that reducing radiation damage in clinical practice can be achieved using a biological approach.

## INTRODUCTION

Diagnostic medical examination exposure accounts for a large fraction of annual radiation exposure (~65% in Japan and ~20% worldwide) [1]. Notably, several reports have shown that computed tomography (CT) examinations increase the risk of cancer (e.g. leukemia and brain tumors) in children and young adults [2, 3]. Neuroendovascular therapy and cardiovascular therapy sometimes induce a tissue reaction (e.g. epilation or erythema) in patients [4, 5]. Therefore, a reduction in the exposure dose received in medical examinations is required. However, in radiodiagnosis, a trade-off between image quality and radiation exposure is inevitable [6]. Insufficient radiation exposure causes blocky image quality that is not acceptable for diagnosis.

Ionizing radiation damages DNA via direct and indirect effects. Approximately 60–70% of damage is induced by an indirect effect that is mediated by reactive oxygen species and/or radicals in low linear energy transfer radiation (e.g. X-rays and γ-rays) [7, 8]. Many reports have shown that cell or mouse death is reduced by treatment of radical scavengers before irradiation [8–10]. However, few studies have investigated the effect of a reduction in radiation damage using radical scavengers in the clinical setting.

Ascorbic acid (AA) is a well-known, safe, anti-oxidant molecule and it is commonly used in clinical sites [11]. AA has anti-oxidant ability, as well as anti-inflammatory [12, 13] and anti-cancer abilities [14]. Notably, AA reduces radiation-induced DNA damage [15, 16] and cell senescence [17], and improves survival in mice [18, 19] and cells [20].

In the present study, we administered AA to patients before cardiac catheterization (CC) for diagnostic purposes. We analyzed changes in the number of phosphorylated H2AX (γH2AX) foci (a marker of DNA double-strand breaks) in lymphocytes, red blood cell (RBC) glutathione levels, blood cell counts, and biochemical parameters.

## MATERIALS AND METHODS

### Patients

We analyzed nine patients who had CC for diagnostic purposes from January 2016 to March 2016. The patients were randomly selected, and were randomly divided into control (Patients 1–5) and AA (Patients 6–9) arms.

This study was conducted in accordance with the Declaration of Helsinki. All protocols were approved by the institutional review board of the Social Insurance Nogata Hospital (Nogata, Fukuoka, Japan). Written informed consent was obtained from the patients before commencing procedures.

### Angiographic technique

For CC, a biplane X-ray imaging system (INFX-8000 V/JB, Toshiba, Japan) was used. The dose–area product (DAP) [21], cumulative dose at the interventional reference point (CD-IRP) [21], fluoroscopic time, number of series, number of total frames, number of cone beam CTs, and working angle were recorded.

### Study design

**Fig. 1.**
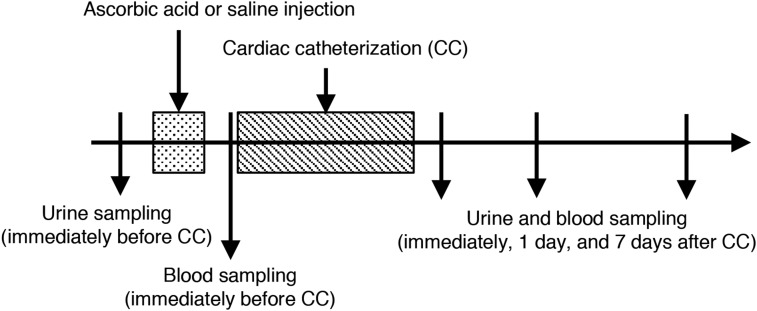
Treatment and sampling schedule. The patients were randomly divided into the control and ascorbic acid (AA) arms. AA (or saline) was administered intravenously in a hospital room after urine sampling. Patients were transferred to the angio room and had blood samples collected before they underwent cardiac catheterization (CC). After CC, patients returned to the hospital room, and had blood and urine sampling performed. Patients had blood and urine sampling at 1 day and 1 week after CC.

The study design is shown in Fig. 1. A volume of 20 mg/kg AA (Towa Pharmaceutical Co. Ltd, Osaka, Japan) (or saline for the control group) was administrated intravenously in a hospital room after urine sampling. This dose was decided upon on the basis of a Japanese interview form (maximum dose of 2 g once daily). After AA treatment, patients were transferred to the angio room and blood was collected before they underwent CC. After CC, patients returned to the hospital room, and blood and urine sampling was performed. Patients also had blood and urine sampling at 1 day and 1 week after CC. Urine was immediately quenched in a dedicated freezer box and stored at −80°C until 8-hydroxydeoxyguanosine (8-OH-dG) measurement. Blood was immediately pretreated for each test.

### Complete blood count

Blood was collected into EDTA-containing tubes. Sample analysis for the complete blood count was performed by SRL Inc. (Tokyo, Japan).

### Biochemical analyses

Serum or plasma was prepared for measurement of AA, vitamin E, β-carotene, arachidonic acid (ArA), eicosapentaenoic acid (EPA), dihomo-γ-linolenic acid, docosahexaenoic acid, monocyte chemoattractant protein (MCP)-1, platelet-activating factor (PFA)-acetylhydrolase, pentosidine, high-density lipoprotein (HDL)-cholesterol, low-density lipoprotein (LDL)-cholesterol, oxidized LDL, triglyceride, glucose, and hydroperoxide levels, the EPA/ArA ratio, and superoxide dismutase (SOD)-like activity. These parameters were measured by SRL Inc.

### Measurement of the number of γH2AX foci in circulating lymphocytes

The γH2AX fluorescent immunostaining assay was performed as described previously [22, 23].

### Measurement of red blood cell glutathione levels, whole blood anti-oxidant capacity, and 8-hydroxydeoxyguanosine levels

Measurement of RBC glutathione levels was performed using an oxidative glutathione (GSSG)/reduced glutathione (GSH) Quantification Kit (Dojindo, Kumamoto, Japan) as described previously [24]. Measurement of whole blood anti-oxidant capacity was performed using i-STrap (Dojindo) as described previously [24]. Measurement of 8-OH-dG levels was performed using the high-performance liquid chromatography method as described previously [25].

### Statistical analysis

The mean and standard deviation (SD) were calculated for each data point. The Mann–Whitney U test was used to analyze the statistical significance of differences between groups. Pearson’s correlation coefficient test was used to analyze the significance of correlation coefficients. Analysis of covariance was used to analyze the difference in the regression line slope between the two arms. Values of *P* < 0.05 were considered to indicate statistical significance.

## RESULTS

The patients’ characteristics and angiographic parameters are shown in Supplementary Table 1. None of the patients’ characteristics and angiographic parameters showed significant differences between the control and AA arms (Supplementary Table 1).

**Fig. 2.**
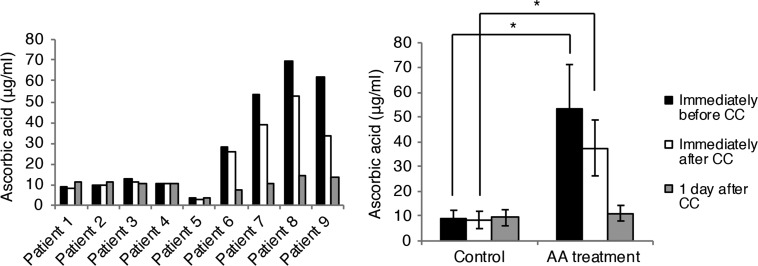
Serum ascorbic acid levels in patients. Individual (left panel) and mean (right panel) serum ascorbic acid (AA) levels in the control and AA arms. Data are presented as the mean ± SD. *P < 0.05, Mann–Whitney U test.

The AA arm showed 5.8 times higher AA levels immediately before CC (*P* < 0.05) and 4.4 times higher AA levels immediately after CC (*P* < 0.05) compared with the control arm. AA doses returned to control levels at 1 day after CC (Fig. 2). No AA treatment-related adverse events were observed.

**Fig. 3.**
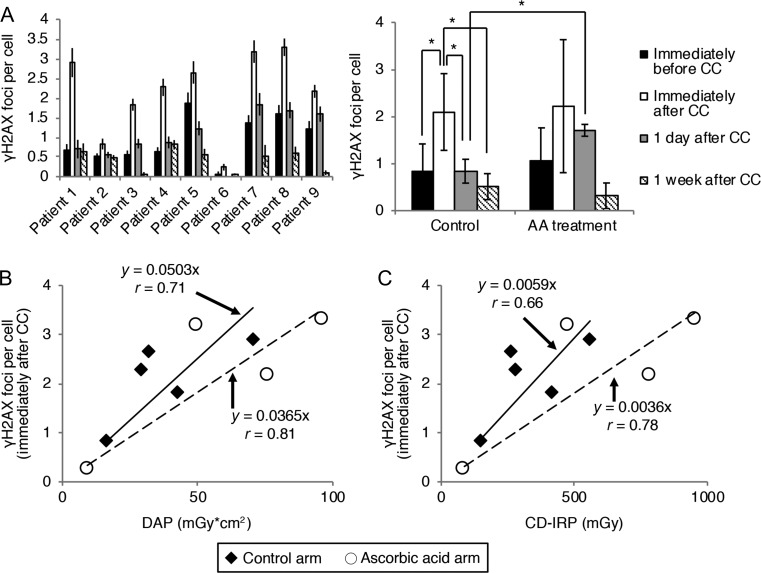
Number of phosphorylated H2AX foci in circulating lymphocytes. (A) Individual (left panel) and mean (right panel) number of phosphorylated H2AX (γH2AX) foci in the control and ascorbic acid (AA) arms. (B, C) Correlation of the number of γH2AX foci immediately after cardiac catheterization (CC) with the exposure dose (dose–area product [DAP], or cumulative dose at the interventional reference point [CD-IRP]). Closed diamonds indicate the control arm and open circles indicate the AA arm. Solid lines on the graphs indicate linear regression for the control arm. Dashed lines on the graphs indicate linear regression for the AA arm. γH2AX foci were counted in at least 100 cells at each time point. Data are presented as the mean ± SD. *P < 0.05, Mann–Whitney U test and Pearson’s correlation coefficient test.

Figure 3A shows the number of γH2AX foci in circulating lymphocytes. In the control arm, the number of γH2AX foci significantly increased immediately after CC and then decreased to the same as that observed before CC at 1 day after CC. In the AA arm, similar to the control arm, the highest number of γH2AX foci was observed immediately after CC. The number of γH2AX foci then decreased to the same as that observed before CC at 1 week after CC. However, these differences were not significant. Furthermore, we found that the AA arm showed a significantly higher number of γH2AX foci than did the control arm at 1 day after CC (Fig. 3A). Subsequently, we investigated whether the number of γH2AX foci immediately after CC was correlated with DAP or CD-IRP (Fig. 3B, C). The control and AA arms showed a medium to strong correlation between DAP (*r* = 0.71 in the control arm; *r* = 0.81 in the AA arm) and CD-IRP (*r* = 0.66 in the control arm; *r* = 0.78 in the AA arm) (Fig. 3B, C). However, these correlations were not significant. We also analyzed the difference in the regression line slope between the control and AA arms using analysis of covariance. However, we did not find a significant difference in the slope between these arms.

**Fig. 4.**
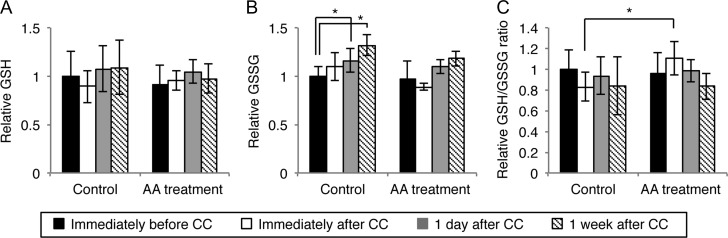
Red blood cell glutathione levels in patients. Mean (A) reduced glutathione (GSH), (B) oxidized glutathione (GSSG), and (C) GSH/GSSG ratio values in the control and AA arms. All quantitative data are presented as the mean ± SD. *P < 0.05, Mann–Whitney U test.

Figure 4 shows RBC glutathione levels. In the control arm, GSSG levels were significantly increased at 1 day and 1 week after CC compared with those immediately before CC. In the AA arm, the GSH/GSSG ratio was higher than those in the control arm immediately after CC. However, these changes were not correlated with DAP and CD-IRP (data not shown). Furthermore, we investigated whether CC or AA treatment affected the numbers of RBCs, white blood cells, lymphocytes, monocytes, and platelets, hematocrit, levels of hemoglobin, vitamin E, β-carotene, ArA, EPA, dihomo-γ-linolenic acid, docosahexaenoic acid, MCP-1, PFA-acetylhydrolase, pentosidine, HDL-cholesterol, LDL-cholesterol, oxidized LDL, triglycerides, glucose, 8-OH-dG, and hydroperoxide, the EPA/ArA ratio, SOD-like activity, and whole blood anti-oxidant capacity (Supplementary Figs 1 and 2). However, none of these parameters were correlated with DAP or CD-IRP (data not shown).

## DISCUSSION

Radiation exposure to patients and sometimes to physicians is a major disadvantage in clinical radiodiagnosis and radiotherapy [21]. This single-center, prospective, randomized, controlled trial was designed to determine whether AA treatment alleviates adverse side effects during CC. AA has low toxicity [11]. Riordan *et al.* reported that only minimal adverse side effects were observed, even at 8 weeks, of 710 mg/kg/day of AA infusion [26]. In our study, we treated patients with 20 mg/kg AA before CC. As expected, we did not observe any AA treatment–related adverse events.

Formation of γH2AX foci in circulating lymphocytes is strongly correlated with the irradiated dose in total body irradiation [27]. In our study, we did not find a significant difference in the number of γH2AX foci immediately after CC between DAP and CD-IRP (Fig. 3B, C). One possible reason for this lack of a significant difference is that the number of patients was too small, which reduced the statistical power. Another possible reason is that lymphocytes circulate in blood. The radiation dose that the lymphocytes received during the examination in our study might have been affected by their circulation, irradiated area, and exposure time. These possibilities should be investigated in future experiments. Unfortunately, we did not find satisfactory evidence to show that AA treatment reduces γH2AX foci formation immediately after CC. In further experiments, we need to increase the number of patients and could consider treating patients with a higher dose of AA. Further, we found that the AA arm had a higher number of γH2AX foci 1 day after CC than did the control arm. This finding suggests that AA treatment leads to complex radiation-induced DNA damage or suppresses DNA repair. In future studies, we need to directly confirm DNA damage using the comet assay and analyze the kinetics of DNA repair.

The GSH/GSSG ratio is a marker of the cellular redox state [28]. Sun *et al.* and Navarro *et al.* reported that RBCs or the whole blood GSH/GSSG ratio was decreased after irradiation [24, 29]. In this study, we found that GSSG levels were significantly increased at 1 day and 1 week after CC compared with before CC in the control arm. However, these changes in glutathione were not correlated with the exposure dose (DAP and IRP-CD). Therefore, we cannot conclude whether these changes were induced by radiation. Furthermore, the control arm showed a significantly lower GSH/GSSG ratio than did the AA arm immediately after CC. This finding suggests that AA treatment weakly alleviates oxidative stress after CC.

Additionally, we analyzed the complete blood count and 19 biochemical parameters (Supplementary Fig. 1). However, we did not find any significant differences in these parameters between before and after CC. Several parameters (e.g. RBCs, hemoglobin, docosahexaenoic acid) were slightly decreased immediately after CC in the control and AA arms. These changes were probably due to bleeding in CC. Triglyceride levels were also decreased immediately after CC in the control and AA arms, which could have been due to the long interval between blood sampling immediately after CC and when patients had a meal. The AA arm showed lower hydroperoxide levels and a trend for higher SOD-like activity than did the control arm before and after CC (Supplementary Fig. 1). These changes were likely due to AA treatment.

Our study has some limitations. First, we only analyzed nine patients, for various reasons, which reduced statistical power. A larger-scale, prospective, multicenter study is required in the future. Second, AA treatment showed only a limited effect in the present study. Therefore, a higher AA dose needs to be examined. Feliciano *et al.* showed that redox nanoparticles effectively reduced organ dysfunction and death in irradiated mice [9]. These novel anti-oxidants should also be estimated in the clinic. Third, we reported data that do not equate to the risk of cancer. Further studies should analyze carcinogenesis-related parameters, such as chromosomal aberration [30] and the micronuclei [31]. Fourth, many studies have reported that radiation increases mitochondrial reactive oxygen species production [32, 33]. This oxidative stress induces DNA damage and genome instability [34, 35]. These reports indicate that sustained anti-oxidant treatment may be able to more effectively reduce radiation-induced side effects than a single treatment. Indeed, Ito *et al.* reported that pre- and post-treatment of AA significantly improved radiation-induced gastrointestinal damage compared with pre-treatment of AA in mice [19]. Fifth, the present study only focused on diagnostic procedures and did not include therapeutic procedures. Therapeutic coronary or neurovascular interventions cause tissue reactions (deterministic effects; e.g. erythema, epilation and cataracts) [4, 5, 36]. Further studies should include therapeutic procedures and investigate whether anti-oxidant treatment reduces tissue reactions. Sixth, CC is an essential procedure for precise diagnosis of and/or treatment for congenital cardiac diseases. These patients undergo multiple CCs from newborns to adolescents and young adults, resulting in an increase in radiation risk [37, 38]. A reduction in radiation damage is urgently required in these patients.

In conclusion, although our sample size was small, we performed an interventional clinical study of AA treatment in CC. This approach is unique in that it uses radical scavengers to attempt to reduce diagnostic radiation exposure–induced damage. This approach is challenging, but it could be a novel strategy for reducing radiation damage in clinical practice using a biological approach.

## Supplementary Material

rrz038_2018igarashi-SpTable1-sun3-E2

rrz038_SpFig
