# Supplementary material for: Pilot clinical study of ascorbic acid treatment in cardiac catheterization
Source: J Radiat Res. 2019 Jun 28;60(5):573–8. doi: 10.1093/jrr/rrz038 (PMC6805981; doi:10.1093/jrr/rrz038)

Supplementary Fig. 1
(complete blood count)

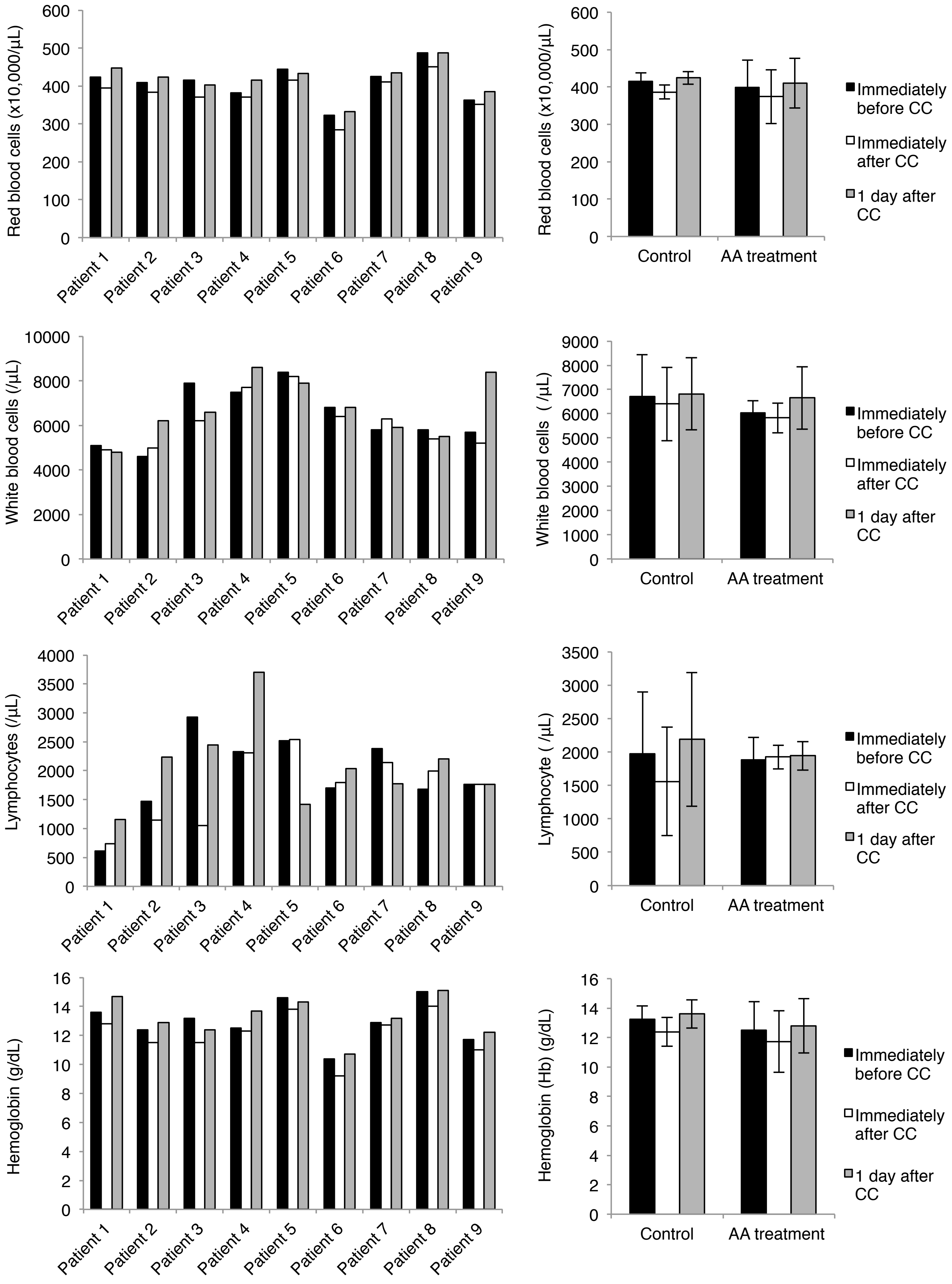

Supplementary Fig. 1
(complete blood count)

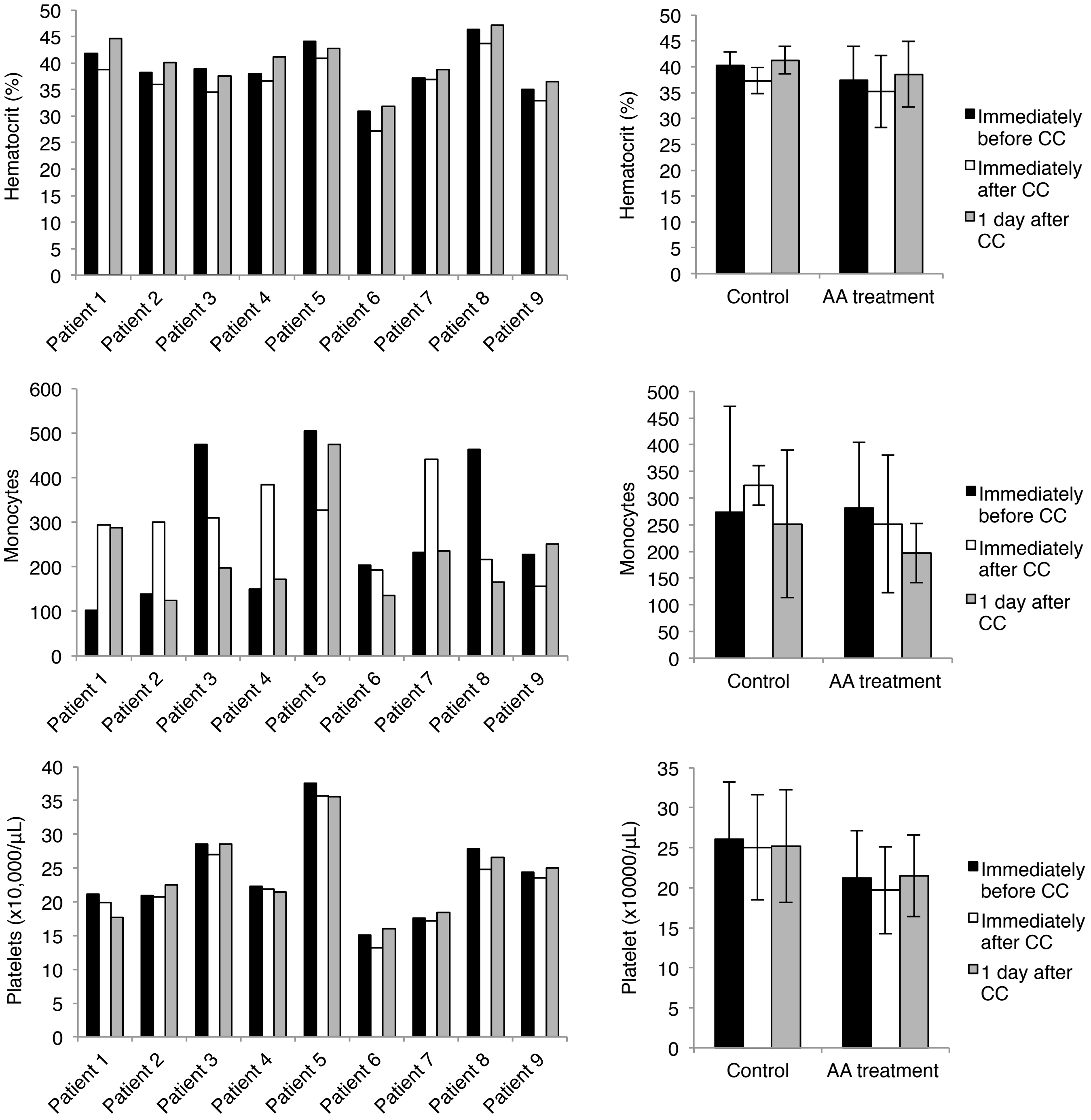

Supplementary Fig. 1
(vitamins)

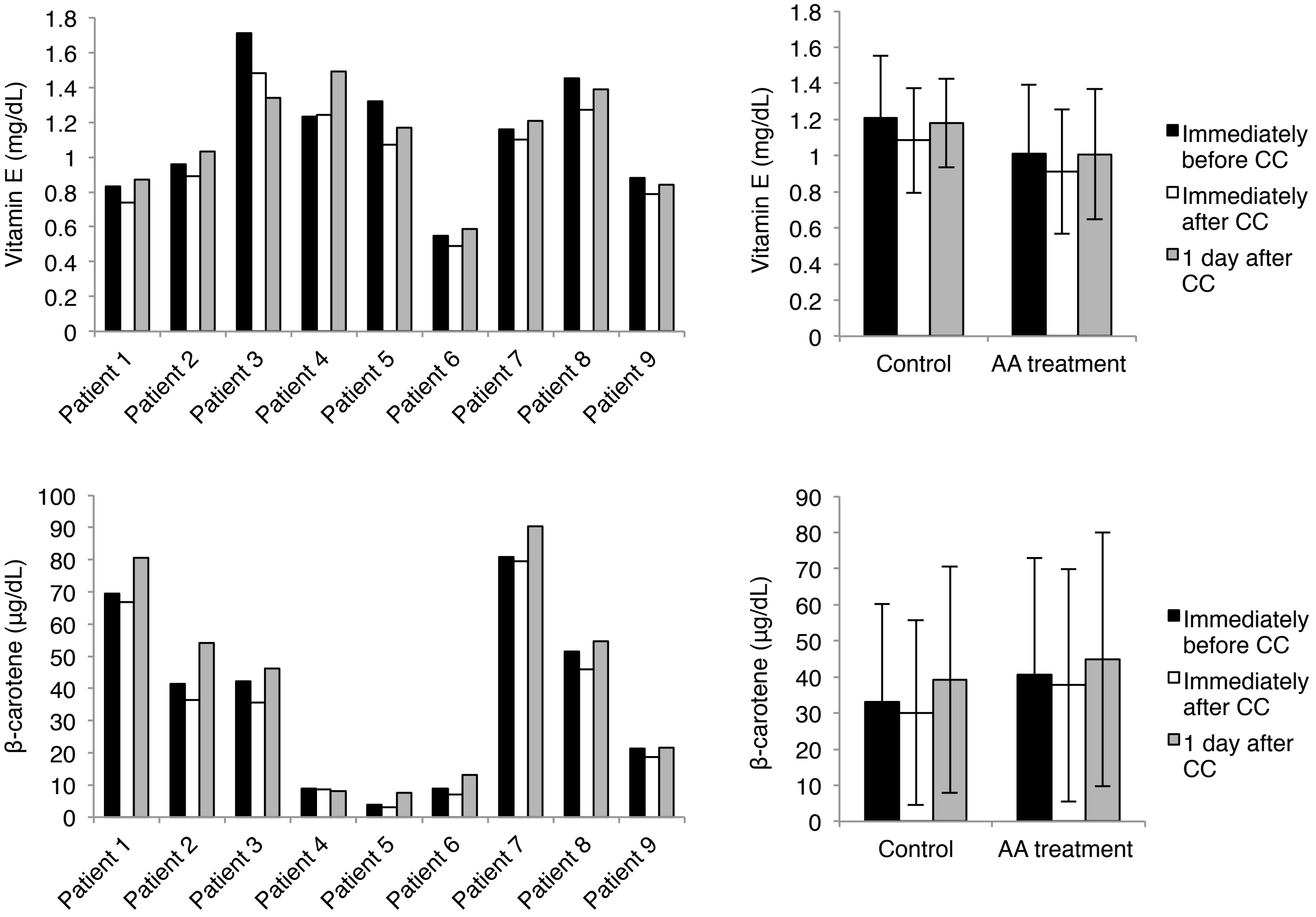

Supplementary Fig. 1
(amino acids)

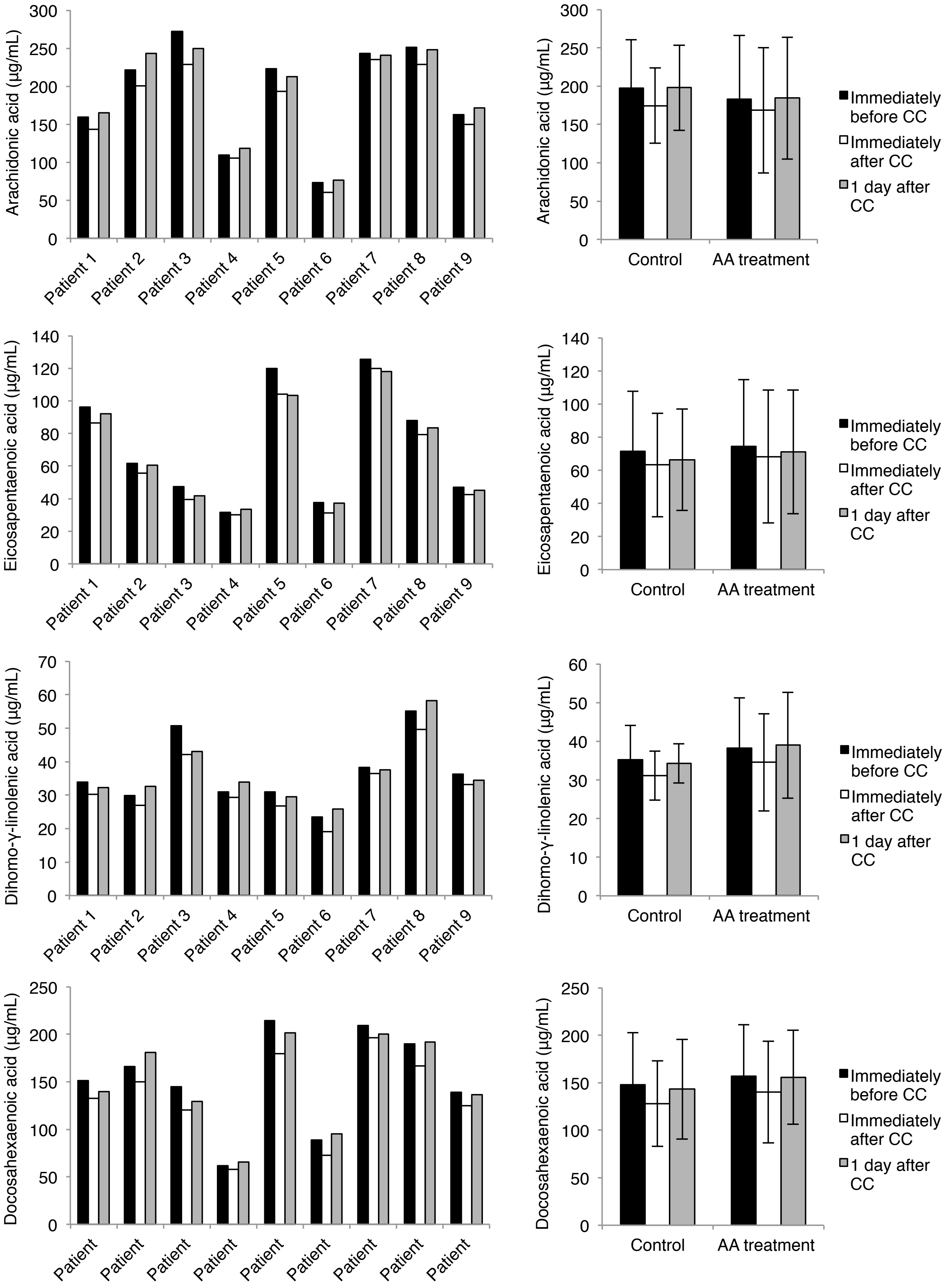

Supplementary Fig. 1
(enzymes)

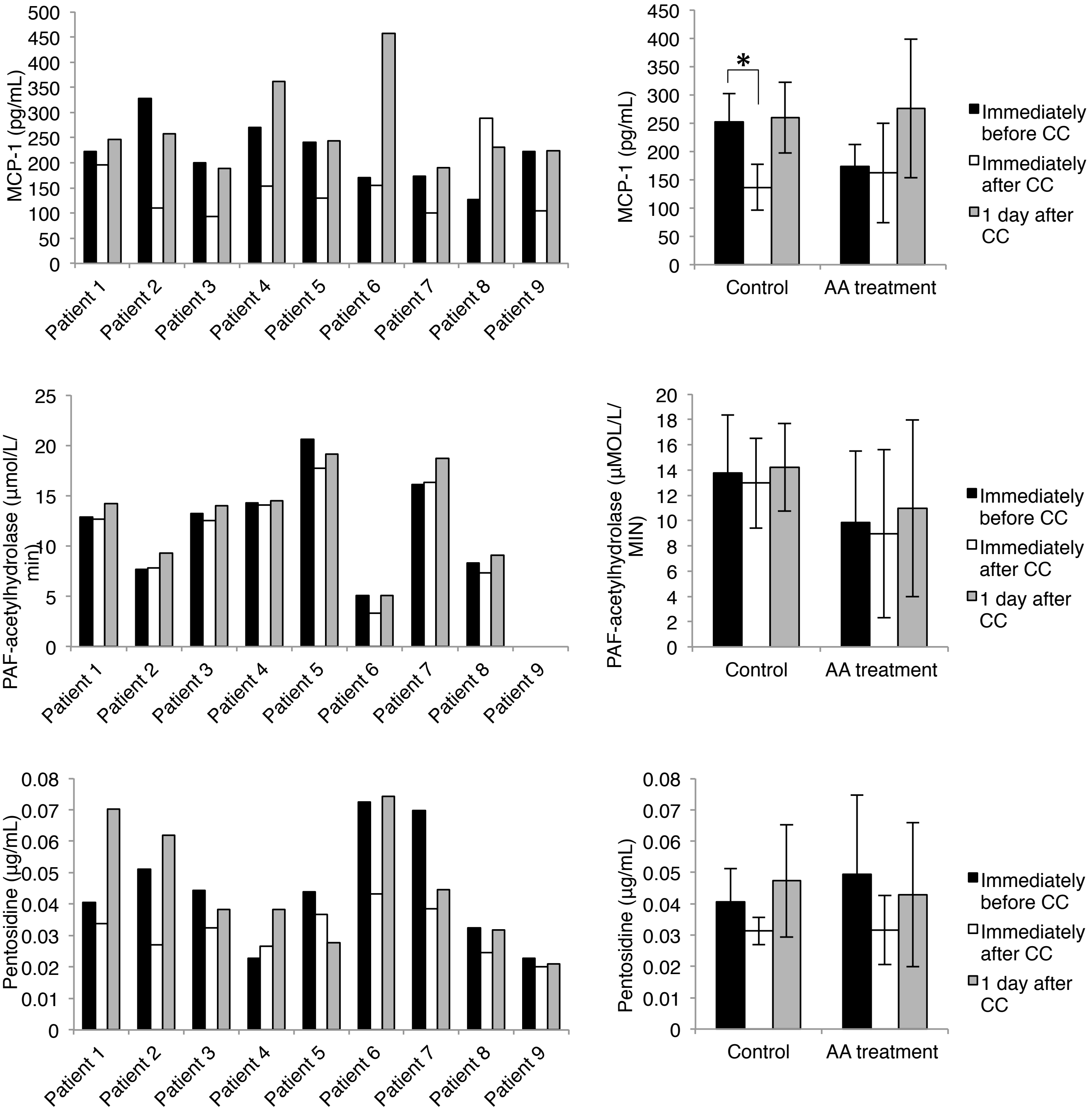

Supplementary Fig. 1
(lipids)

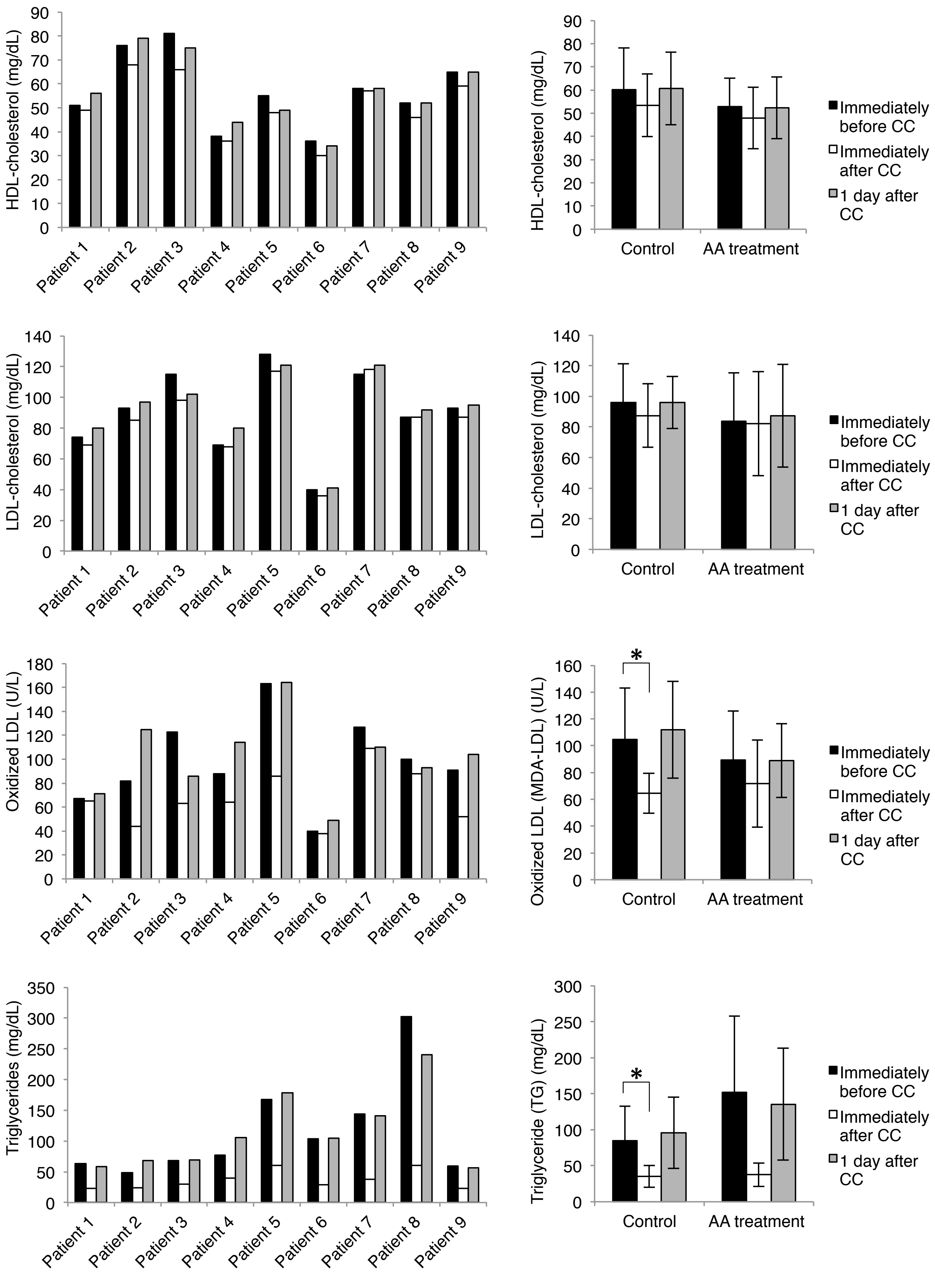

Supplementary Fig. 1
(metabolic parameters)

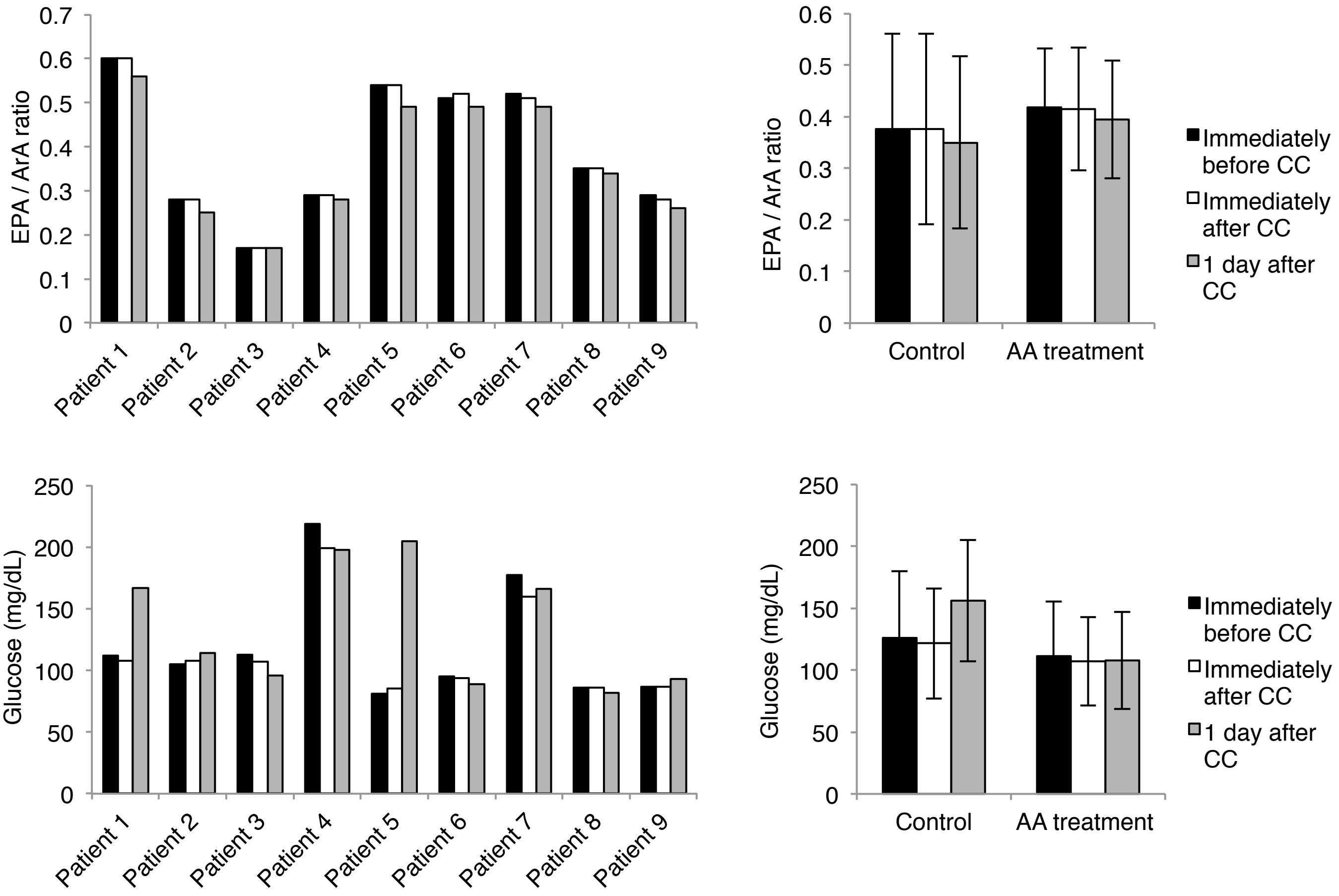

Supplementary Fig. 1
(redox parameters)

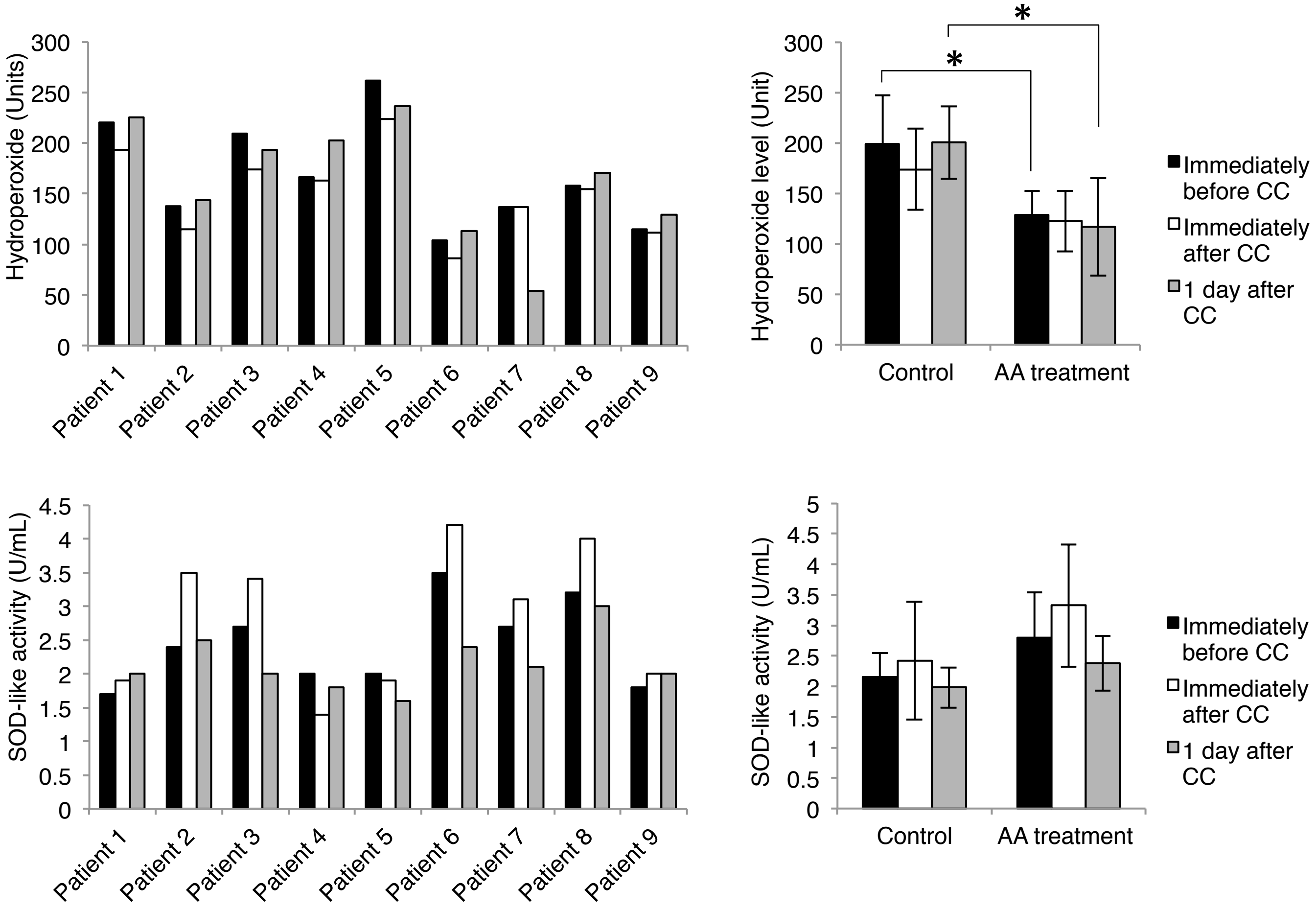

Supplementary Fig. 2

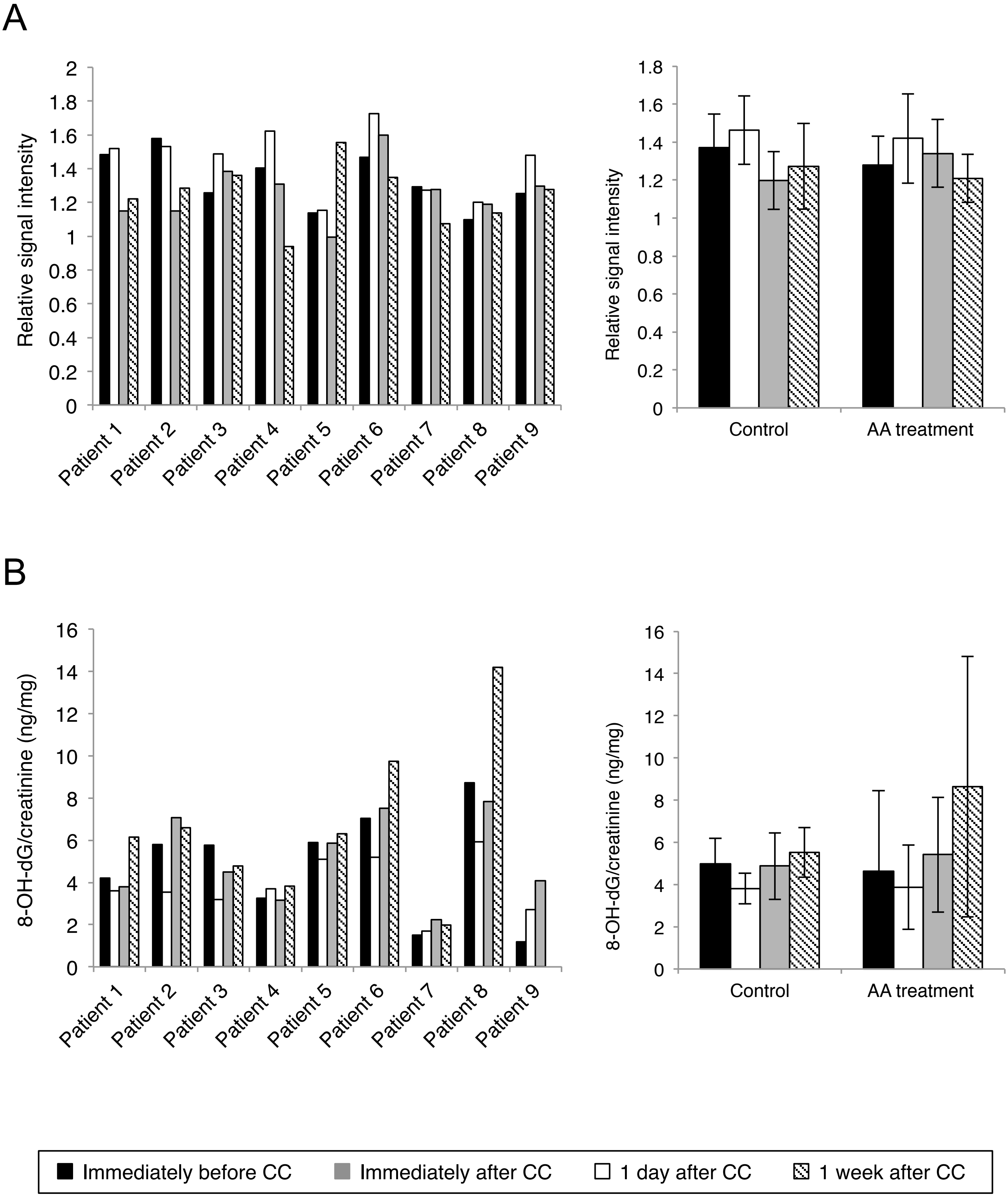

Supplement: rrz038_SpFig [file rrz038_spfig.pdf]
